# The New Human *Babesia* sp. FR1 Is a European Member of the *Babesia* sp. MO1 Clade

**DOI:** 10.3390/pathogens10111433

**Published:** 2021-11-04

**Authors:** Claire Bonsergent, Marie-Charlotte de Carné, Nathalie de la Cotte, François Moussel, Véronique Perronne, Laurence Malandrin

**Affiliations:** 1 BIOEPAR, INRAE, Oniris, 44300 Nantes, France; nathalie.delacotte@oniris-nantes.fr; 2 Service de Maladies Infectieuses et Tropicales, Hôpital F. Quesnay, 78200 Mantes-la Jolie, France; mcdecarne@ch-versailles.fr (M.-C.d.C.); veronique.perronne@aphp.fr (V.P.); 3 Laboratoire de Biologie Médicale, Hôpital F. Quesnay, 78200 Mantes-la-Jolie, France; f.moussel@ch-mantes-la-jolie.fr

**Keywords:** *Babesia divergens*, *Babesia* sp. MO1, *Babesia capreoli*, *rap-1a*, *ama-1*, phylogeny

## Abstract

In Europe, *Babesia divergens* is responsible for most of the severe cases of human babesiosis. In the present study, we describe a case of babesiosis in a splenectomized patient in France and report a detailed molecular characterization of the etiological agent, named *Babesia* sp. FR1, as well as of closely related *Babesia divergens*, *Babesia capreoli* and *Babesia* sp. MO1-like parasites. The analysis of the conserved 18S rRNA gene was supplemented with the analysis of more discriminant markers involved in the red blood cell invasion process: *rap-1a* (rhoptry-associated-protein 1) and *ama-1* (apical-membrane-antigen 1). The *rap-1a* and *ama-1* phylogenetic analyses were congruent, placing *Babesia* sp. FR1, the new European etiological agent, in the American cluster of *Babesia* sp. MO1-like parasites. Based on two additional markers, our analysis confirms the clear separation of *B. divergens* and *B. capreoli*. *Babesia* sp. MO1-like parasites should also be considered as a separate species, with the rabbit as its natural host, differing from those of *B. divergens* (cattle) and *B. capreoli* (roe deer). The natural host of *Babesia* sp. FR1 remains to be discovered.

## 1. Introduction

Babesiosis is a tick-borne disease affecting a wide range of vertebrates worldwide. Symptoms of this disease are caused by the intraerythrocytic development of Protozoa of the genus *Babesia*, causing fever, jaundice, hemoglobinuria and anemia, possibly leading to death, depending on the *Babesia* species and the host. About one hundred species of *Babesia* have been described and transmission of the parasite between hosts occurs almost exclusively through Ixodid tick bites [1]. 

Even though humans are not natural hosts of *Babesia*, human infections caused by several different species of *Babesia* have been reported worldwide. *Babesia microti, B. duncani* (WA1) [2] and to a lesser extent *B. divergens*-like (*Babesia* sp. MO1 clade) [3] have been reported to cause disease in humans in the USA. The most prevalent species is *B. microti* responsible of infections that follow a relatively benign course [4]. In Asia, a few cases have recently been reported, caused by *B. divergens*-, *B. venatorum*- or *B. crassa*-like strains [5,6,7]. 

In Europe, the first case of human babesiosis was described in 1957 in Croatia [8,9]. In 1997, a review on human babesiosis in Europe reported 24 cases in splenectomized (20/24) and non-splenectomized (4/24) patients, 46% of which were fatal even in non-splenectomized patients (2/4) [10]. At that time, the molecular diagnosis of the parasite species was lacking and cases were attributed to *B. divergens* based on morphological and/or serological grounds. A few years later, molecular analysis revealed a new etiological babesiosis agent, *Babesia* sp. EU1, which was found to be responsible for human cases in Austria, Italy [11], Germany [12] and Sweden [13]. Human babesiosis cases due to *B. microti* have been reported in Europe but usually they are imported cases from the USA [14], with only one autochtonous case reported to date in Germany [15]. Severe sporadic cases are usually attributed to *B. divergens* [16,17,18,19,20,21,22,23,24]. However, molecular confirmation of the species is not always undertaken [25,26,27]. Serological analysis and morphology on smears are not sufficient to ascertain *B. divergens* as the etiological agent. Even for specialists, the morphological distinction of *B. divergens* from *Babesia* sp. EU1 on smears is impossible [11,12]. Confirmed cases of babesiosis due to *B. divergens* can remain serologically negative [28,29,30], and serology can be confusing due to dot-like reactivity patterns of most human positive sera, concentrated at the apical pole of the parasite [31]. This reactivity pattern was confirmed with a serum from a clinically and molecularly confirmed human *B. divergens* case in Finland [18,31]. 

The phylogenetic group including *B. divergens* gathers different named or as yet unnamed species that are very closely related, and we will refer to this group as *B. divergens*-like. *B. divergens* is indeed closely related and can be confused with *B. capreoli*, a parasite frequently found in roe deer in Europe, due to their high 18S rRNA sequence relatedness [32,33]. However, the conservation of three base differences in this gene between isolates of *B. divergens* (pathogen of cattle/humans) and isolates of *B. capreoli* (pathogen of roe deer), linked to different in vitro host ranges, allowed the delineation of these two species [33]. *Babesia* sp. MO1 also belongs to this phylogenetic group, and is responsible for a small number of severe or fatal human babesiosis in splenectomized patients in the USA [3,34,35,36]. Cottontail rabbits are the natural hosts of *Babesia* sp. MO1 [37,38]. In vitro cultivation features as well as in vivo experimental infections demonstrated the incapacity of this genetic variant to infect cattle, and, combined with 18S rRNA sequence differences, led to its species differentiation from *B. divergens* and the provisional name *Babesia* sp. MO1 [39,40,41].

In splenectomized patients, babesiosis due to *B. divergens* is fulminant with symptoms that appear within 1–3 weeks post infection, with persistent high fevers and headaches, followed by severe intravascular hemolysis, hemoglobinuria, and jaundice. Babesiosis in splenectomized patients is often fatal in Europe, as diagnosis and therefore adequate treatment are often delayed due to uncharacteristic flu-like symptoms and the infrequency of cases [42,43]. Severe symptoms and fatal cases also occur in non-splenectomized patients with known or unknown predispositions such as splenic dysfunction or a rudimentary spleen [18,19]. In immunocompetent patients, *B. divergens* infection is associated with flu-like symptoms shortly after a tick bite [29] or may remain asymptomatic [44].

In the present study, we describe an unusually mild babesiosis in an asplenic patient in France, originally suspected to be caused by *B. divergens*. Intrigued by the unusual course of infection, we carried out the molecular characterization of the responsible agent. As the 18S rRNA gene is rather conserved within the *B. divergens* taxonomic group, and therefore not sufficiently informative, we supplemented the molecular description with two additional and more variable markers: the apical membrane antigen 1 (*ama-1*) and the rhoptry-associated-protein-1a (*rap-1a*) genes. Molecular characterization and polymorphism of these two genes were also analyzed for different members of the *Babesia divergens*-like phylogenetic group, including the phylogenetically closely related *B. capreoli* and *Babesia* sp. AR1 identical to *Babesia* sp. MO1 but from a patient in Arkansas [35], and compared to available sequences of these genes for *B. divergens*. 

## 2. Results

### 2.1. Babesia *sp*. FR1: Report of the Clinical Case

A 56-year-old man came into the emergency room with a suspected meningitidis syndrome. He was Caucasian and his only notable antecedent was a splenectomy in 2001 following a skiing accident (pneumococcal vaccine administered in November 2016, no *Haemophilus* nor meningococcal vaccines). 

The patient stayed on the Île de Ré from 4 August 2017 to 24 August, then from 29 August to 3 September, at a house located at the edge of a forest. He also stayed in Béthune from 25 August to 28 August. He had a fever for 2 weeks associated with headaches. He then developed severe asthenia, sweats, tachypnea, myalgia, and elbow, shoulder, and knee arthralgia. 

The first blood test (5 September) revealed thrombopenia: 99 giga/L, CRP 65.6 mg/L, ASAT 82U/L, ALAT 73U/L. On 9 September 2017, he developed vomiting, photophobia and a stiff neck, which led to the patient being transferred to hospital (11 September). Nothing specific was revealed by non-injected brain CT. Lumbar puncture was normal, and culture was sterile. Blood tests revealed the following: platelets 71 giga/L, leukocytes 8.40 giga/L (PNN 7.056 giga/L, lymphocytes 0.670 giga/L), hemoglobin 14.4g/dL, ASAT 57 U/L, ALAT 61 U/L, GGT 128 U/L, PAL 188 U/L, normal kidney function. 

**Figure 1:**
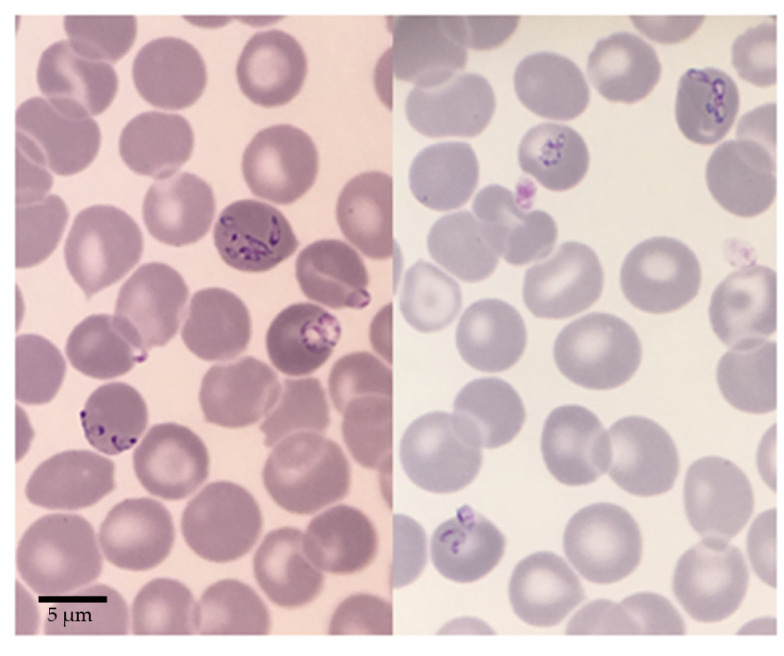
Blood smears of *Babesia* sp. FR1 used to diagnose the *Babesia divergens*-like infection of the patient. Human red blood cells infected with dividing pear shaped merozoites are visible, as well as rounded trophozoites. Bar = 5 µm.

On 12 September, when admitted to the infectious disease unit, clinical examination showed fever, asthenia, and non-significant axillary lymph nodes. The same day, a blood smear showed red blood cells with *Babesia* corpuscles inside, reaching a parasitemia of 3.7% (Figure 1). Blood analysis revealed thrombopenia (60 giga/L) and hemolysis signs without anemia (Hb 14.4 g/dL, LDH 890 U/L). Lyme, HCV and HBV serologies were all negative, and protein electrophoresis was normal. 

Antimicrobial therapy was undertaken on the same day with Atovaquone (750 mg/12h) and Azithromycine (500 mg on day 1 then 250 mg per day). The patient rapidly felt better with apyrexia and disappearance of all symptoms. On 14 September, parasites were still detected on the blood smear and cytolysis was persistent. 

Diagnosis of a *B. divergens*-like infection was confirmed by serology (IFAT with *B. divergens* antigen) with a titer of 1:1024 and by PCR on the 18S rRNA gene as described in materials and methods. Sequencing of the amplified 18S rRNA gene portion confirmed that the responsible agent was closely related to *B. divergens*, the most commonly responsible agent of human babesiosis in France, but different. 

Control of the patient’s infectious status was performed 16 months later. Serology using the same antigen remained positive with a titer reduced to 1:128. PCR was negative. 

### 2.2. Analysis of 18S rRNA Sequences and Position of Babesia *sp*. FR1 within the B. Divergens-Like Phylogenetic Group

A 1641 bp sequence was obtained for *Babesia* sp. FR1, covering the positions that are discriminant among members of the *B. divergens*-like phylogenetic group: nucleotide positions 631, 663, 819, and 1637. The sequence is highly similar (99.95%) to published *Babesia* sp. MO1-like and *B. capreoli* sequences, with only one nucleotide modification at position 819 and 663 respectively. It is also related to *B. divergens* (99.9%) with two nucleotide substitutions at positions 631 and 1637 (Table 1). 

The sequence of 1643 bp from the Arkansas case [35] obtained in this study (named *Babesia* sp. AR1) was 100% identical to the first *Babesia* sp. MO1 case from Missouri (GenBank AY048113) [3], to the Kentucky case (GenBank AY887131) [34] and to the cottontail rabbit isolates [38]. They differ from *B. divergens* by three mutations at positions 631, 819 and 1637 (99.8% identity), and from *B. capreoli* by two mutations at positions 663 and 819 (99.9% identity) (Table 1). 

### 2.3. Major Differences in Ama-1 and Rap-1a Genes within the B. divergens-Like Phylogenetic Group

**Figure 2:**
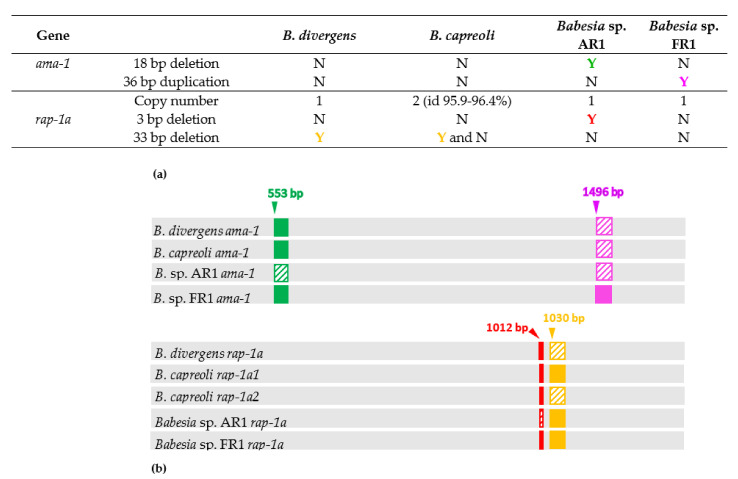
Major differences in *ama-1* and *rap-1a* genes between members of the *B. divergens*-like phylogenetic group. (**a**) Copy number, and presence (Y) or absence (N) of insertion/deletion in *ama-1* and *rap-1a* genes; (**b**) Schematic representation of the major differences and their positions in gene sequence. The colors used in the part (**a**) correspond to the colors used in the graphical representation of the corresponding deletions/insertions in the part (**b**). The deleted or absent regions are dashed.

Before analyzing the detailed sequence polymorphism of *ama-1* and *rap-1a* genes between the *B. divergens*-like phylogenetic group members, some major differences in gene sequences appeared on the alignment (Figure 2).

Complete *ama-1* sequences (sizes between 1803 and 1857 bp) were obtained in this study for four clonal lines of *B. capreoli*, for *Babesia* sp. FR1 and for *Babesia* sp. AR1, and were compared to *B. divergens ama-1* sequences [45]. An 18 bp sequence located between bases 553 and 570 was absent only in *Babesia* sp. AR1, corresponding probably to a deletion, which did not modify the translation frame (Supplementary Figure S1). *Babesia* sp. FR1 *ama-1* sequence differs from all the others by an insertion of a 36 bp sequence located between bases 1496 and 1531. The inserted sequence is highly similar to an upstream 36 bp sequence (differing by two nucleotides) and seems therefore to correspond to a gene conversion event of this small gene portion.

Regarding the *rap-1a* gene, partial sequences (sizes between 1203 and 1236 bp) were obtained for four clonal lines of *B. capreoli*, for *Babesia* sp. FR1 and for *Babesia* sp. AR1, and were compared to *B. divergens rap-1a* sequences [46]. Comparison of *rap-1a* sequences from *B. divergens*, *Babesia* sp. AR1, and *Babesia* sp. FR1 highlighted a 3 bp deletion (nucleotides 1012 to 1014) in *Babesia* sp. AR1 only and a 33 bp deletion located between bases 1034 and 1066 in the 12 *rap-1a B. divergens* sequences performed, which did not modify the translation frame. As superposed chromatograms were observed for *B. capreoli* at the 3′ end of the *rap-1a* gene, the presence of multiple copies of this gene was suspected and confirmed by cloning/sequencing and subsequent specific amplifications of each gene copy from each of the four isolates. Two *rap-1a* gene copies were observed, which we named *rap-1a1* and *rap-1a2*. The *rap-1a1* copy was characterized by the absence of the two deletions, as found for *Babesia* sp. FR1. The *rap-1a2* copy contained the 33 bp deletion only and therefore resembled *B. divergens rap-1a*.

These major deletions/insertions were not included in the sequence identity calculations, nor in the phylogenetic analyses, as they represent one-time, usually non-reversible events, with different evolutionary tempo and mode compared to substitutions.

### 2.4. Intraspecific Sequence Diversity of Rap-1a and Ama-1 within B. divergens and B. capreoli

The genetic variability within the *B. divergens* and *B. capreoli* strains have been analyzed previously for the 18S rRNA gene and no variations were found [33]. 

Intraspecific genetic variability of *B. divergens ama-1* and *rap-1a* genes was previously analyzed in studies performed at our lab and was found to be very low [45,46]. Sequence identities higher than 99.5% were highlighted for both genes, when comparing sequences of the same set of nine and twelve French isolates, for *ama-1* and *rap-1a* respectively (Table 2 and Table S1). The *ama-1* sequences showed between 99.9 to 100% conserved sites; two similar nucleotide substitutions were noted in *ama-1* sequences of 1505B F14, 3601B E2 and Rouen87 F5 isolates, compared to *ama-1* sequences of the other six clonal lines. The *rap-1a* sequences showed sequence identities between 99.6 and 100%, corresponding to a pairwise maximum of six nucleotide substitutions. 

In the case of *B. capreoli*, we analyzed the genetic polymorphism of *ama-1* and *rap-1a* for four isolates collected and cultivated at our laboratory from previous studies [33,47]. The *ama-1* sequences showed sequence identities between 99.7 to 100%. The 2770 F6 and CVD08 005 *ama-1* sequences were identical and up to nine polymorphic sites were identified resulting in six different nucleotide substitutions between *ama-1* sequences of 2704C and 2801 F10 isolates, and were compared to the other two identical *ama-1* sequences.

The two copies of the *rap-1a* gene (*rap-1a1* and *rap-1a2*) were identified in all four *B. capreoli* isolates. Sequence variability of each gene copy was low (less than seven nucleotide substitutions), and identities ranged between 99.5–100% and between 99.6–100% among the *rap-1a1* and the *rap-1a2* sequences, respectively. Sequence identities between *rap-1a1* and *rap-1a2* copies ranged between 96.1 and 96.5%. Most substitutions specific to each gene copy (39 positions) were non silent (39 substitutions resulting in 28 amino acid modifications), with a majority of substitutions on the first (nine substitutions) and/or second codon position (16 substitutions). 

### 2.5. Genetic Variability within the B. divergens-Like Phylogenetic Group

As explained above, sequence identities were calculated without the regions corresponding to deletions/insertions and are presented as a contingency table including all three analyzed genes (Table 3). In general, the *ama-1* gene seemed to be more conserved than the *rap-1a* gene as the percentage of sequence identities ranged between 94.3 to 98.7% for *ama-1* and between 86.6 to 98.7% for *rap-1a*. For both genes, the lowest sequence identities were evidenced between *B. divergens* and all other analyzed *Babesia* within the group. The highest sequence identities for *ama-1* and *rap-1a* were obtained between *Babesia* sp. FR1 and *Babesia* sp. AR1 sequences (98.7% identities for both genes). *B. capreoli* was found to be more closely related to *Babesia* sp. AR1 and *Babesia* sp. FR1 than to *B. divergens*.

It was not possible to determine if one of the two copies of *B. capreoli rap-1a* was more related to the unique *rap-1a* gene sequence of other members of the phylogenetic group, as sequence identity values were highly similar.

### 2.6. Phylogenetic Analysis

**Figure 3:**
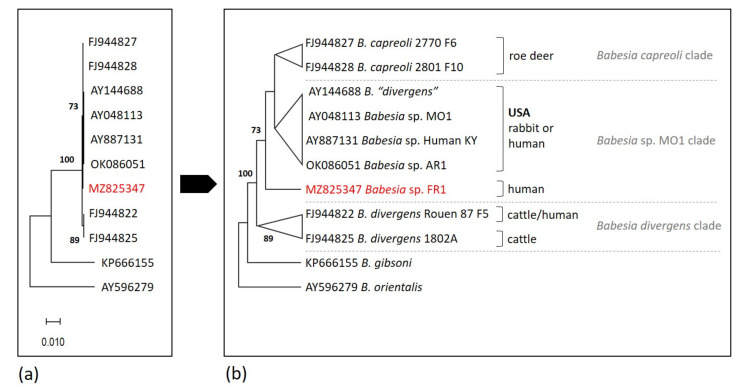
Maximum likelihood unrooted phylogenetic tree of *Babesia* from the *Babesia divergens*-like phylogenetic group based on partial 18S rRNA sequences (1189 bp in the final data set). Branch support/bootstrap values are indicated at each node. *Babesia* sp. FR1 sequence obtained in this study is emphasized in red. (**a**) Scale bar indicates nucleotide substitution rate per site. (**b**) Topology of the tree allowing a better visualization of the bootstrap values; hosts of *Babesia* isolates are indicated.

**Figure 4:**
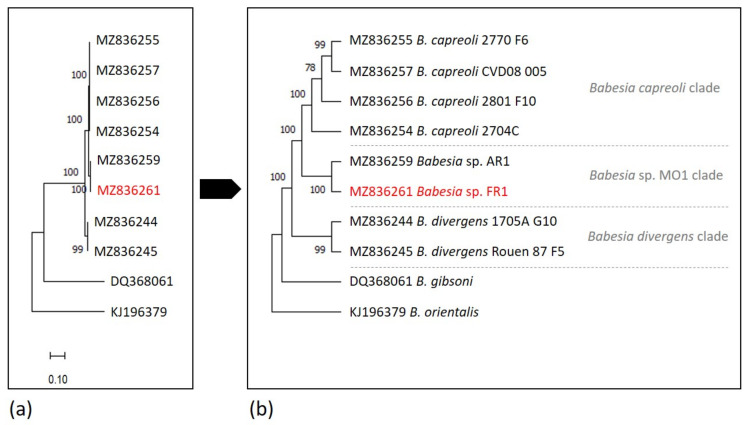
Maximum likelihood unrooted phylogenetic tree of *Babesia* from the *Babesia divergens*-like phylogenetic group based on partial *ama-1* gene sequences (1728 bp in the final data set). Branch support/bootstrap values are indicated at each node. *Babesia* sp. FR1 sequence obtained in this study is emphasized in red. (**a**) Scale bar indicates nucleotide substitution rate per site. (**b**) Topology of the tree allowing a better visualization of the bootstrap values.

**Figure 5:**
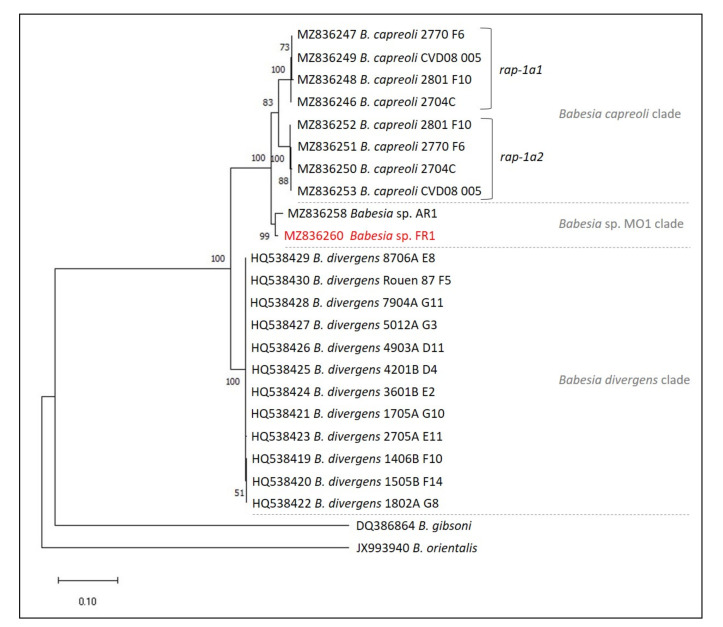
Maximum likelihood unrooted phylogenetic tree of *Babesia* from the *Babesia divergens*-like phylogenetic group based on partial *rap-1a* gene sequences (1138 bp in the final data set). Branch support/bootstrap values are indicated at each node. *Babesia* sp. FR1 sequence obtained in this study is emphasized in red. Scale bar indicates nucleotide substitution rate per site.

The phylogenetic analyses based on 18S rRNA, *ama-1* and *rap-1a* genes were concordant and confirmed the placement of the *Babesia* sp. FR1 into the *B. divergens*-like phylogenetic group, with strong bootstrap values of 100 (Figure 3, Figure 4 and Figure 5). According to the 18S rRNA phylogenetic analysis, and despite the high level of conservation of this marker, two sister groups were supported by good bootstrap values, and *Babesia* sp. FR1 clustered with *B. capreoli*, *Babesia* sp. AR1 and *Babesia* sp. MO1 (bootstrap of 73), and not with *B. divergens* (forming the second cluster supported by a bootstrap value of 89) (Figure 3). The separation of these two clusters was also well supported in the phylogenetic analysis with *ama-1* and *rap-1a* as markers (Figure 4 and Figure 5). The *B. divergens* clade was supported by bootstrap values of 99 and 100 (*ama-1* and *rap-1a* respectively). The *B. capreoli*/*Babesia* sp. AR1/*Babesia* sp. FR1 clade was also well supported by bootstrap values of 100 (*ama-1* and *rap-1a*), but splits on the one hand into a subclade with *B. capreoli* (bootstraps of 100 and 83) and on the other hand into a second subclade with *Babesia* sp. FR1 and *Babesia* sp. AR1 (bootstraps of 100 and 99). The two *Babesia capreoli rap-1a* copies clustered into two sister groups with strong support (100). 

## 3. Discussion

Most human babesiosis cases are recorded in North America and are mainly due to *Babesia microti*, sporadically to *B. duncani* (*Babesia* sp. WA1) and to *Babesia* sp. MO1-like parasites. Sporadic cases were reported in Asia, Africa, and South America, with diverse and often partially characterized etiological agents [48]. In Europe, human babesiosis is rare and *B. divergens* is the main causal agent [43,49]. The most impacted countries are France, Ireland and Great Britain, and in France, Western regions and Normandy are most affected [10], due to substantial farming of bovines, the natural host of *B. divergens* [50]. 

The patient was most probably bitten by a tick on the Île de Ré, even if he had no recollection of a tick bite. This is the most probable place of tick acquisition by the patient, as it is close to a forest, where the abundance of the potential vector *I. ricinus* is high, increasing the risk of contracting tick-borne pathogens [51]. The patient was asplenic, which is also a major risk factor for severe or fatal babesiosis [10,43,49]. However, the symptoms in this patient developed slowly (two to three weeks between the onset of symptoms and admission to hospital) despite aspleny, while *B. divergens’* course of infection in such cases is usually fulminant [42]. Biological diagnosis of the provisionally named *Babesia* sp. FR1 was based on a blood smear, which led to the administration of antibabesial therapy (Atovaquone and Azithromycine) as soon as practicable. This treatment was effective, as symptoms rapidly disappeared, and parasite clearance was attested 16 months later by a negative PCR, correlated with a reduction of the serology titer. 

Molecular characterization of *Babesia* sp. FR1 required a deeper analysis. Sequence and phylogenetic analysis of 18S rRNA revealed that it was genetically close but different from typical *B. divergens* isolates infecting cattle or humans (two polymorphic sites at positions 831 and 1637) [33], but that it closely resembled the American *B. divergens*-like parasite *Babesia* sp. MO1. Despite the genetic difference with *B. divergens*, the infection could be diagnosed using *B. divergens*-specific serological tools (IFAT), confirming anyway a close relationship with *B. divergens.*

We decided to explore new markers to improve knowledge on the *B. divergens*-like phylogenetic clade and to correctly position this new isolate within this species complex. We chose *rap-1a* and *ama-1* for two reasons. First, both genes code for proteins involved in the process of red blood cell invasion by the parasite [52], and as host range/specificity is an important biological feature in the description of this intra-erythrocytic obligatory parasite, they represent markers of interest. Second, we know from previous studies that both genes were well conserved among *B. divergens* isolates from cattle or humans [45,46]. Their interspecies divergence remained to be determined. 

Regardless of the marker used, the sequences of *B. divergens* (cattle as a natural host) are grouped in a cluster well-separated from the other two clusters corresponding to *B. capreoli* and *Babesia* sp. MO1/AR1/FR1. Phylogenies based on more discriminant markers (*rap-1a* or *ama-1*) placed *Babesia* sp. FR1 in the cluster formed by isolates responsible for cases of human babesiosis in the USA represented by *Babesia* sp. AR1. This cluster is separated from the cluster of *B. capreoli* sequences, and from the cluster of *B. divergens* sequences. We can therefore conclude that *Babesia* sp. FR1 is not a *B. divergens*. 

The phylogenetic group containing *Babesia* sp. MO1 is sometimes referred to as the *B. divergens* US lineage [53,54]. However, *Babesia* sp. FR1, which clusters with *Babesia* sp. MO1 and *Babesia* sp. AR1, was clearly acquired locally and is an autochthonous case as the patient did not travel to the USA in the months before the onset of the symptoms. Therefore, we not only confirm in the present study that *Babesia* sp. MO1-like sequences form a well-supported taxon, but we also highlight that the geographical distribution of this group is not restricted to the USA, but extends to Europe as it includes *Babesia* sp. FR1. The three clusters within *Babesia divergens*-like, i.e., *B. divergens*, *B. capreoli,* and *Babesia* sp. MO1-like might be associated with their natural host rather than with geographic distribution. Humans are only incidental hosts for parasites belonging to the *B. divergens*-like group, the natural hosts being cattle for *B. divergens*, roe deer for *B. capreoli* and rabbits for *Babesia* sp. MO1. The natural host for *Babesia* sp. FR1 has not been characterized but could also well be a Laporidae, especially in the Île de Ré context, a highly touristic and populated island where cattle and cervids are rare or absent, due to limited forested areas dominated by resinous trees (mainly maritime pine trees) and typical local productions (vineyards and salt marshes). The European rabbit (*Oryctolagus cuniculus*) is highly abundant on this island where it has been pullulating since the 2000s, and could therefore be the potential natural host for *Babesia* sp. FR1.

In this study, we did not include isolates described as *B. divergens* in sika deer described in Japan or in humans in China [6,53,54]. Our goal in this study was to characterize and correctly place the new *Babesia* sp. FR1 isolate in the phylogenetic group of *Babesia divergens*-like, among biologically well-characterized isolates, i.e., whose host range has been studied and whose parasites have been cultured [33,38,39,40,41,55]. The isolates described in Japan from sika deer and named *B. divergens* are not included in the *B. divergens*-like phylogenetic group because they differ at the 18S rRNA sequence from all other members of this group by at least six conserved substitutions all of which are different from those described within this group. These isolates form a sister group to *B. divergens*-like. The name *B. divergens* should be reserved for isolates from cattle or humans whose 18S rRNA sequences match the many descriptions already published [18,19,20,21,22,23,29,30,33,49]. There is no evidence that isolates from sika deer are capable of infecting either cattle, gerbils (*B. divergens* experimental host), or humans. 

The sequences named as “*B. divergens*” and described in humans in China [6] actually match those described for *B. capreoli*, with the characteristic differences at positions 631 and 663 [33]. This information raises the possibility of human infections by *B. capreoli*, a species that has never been molecularly characterized as responsible for symptomatic cases of human babesiosis in Europe and described as not growing in vitro in human red blood cells [33]. However, short-term asymptomatic carriage of parasites could occur in humans in geographic areas with high parasite and vector prevalences. We unsuccessfully attempted to obtain DNA from these parasites to include them in our study. 

In the present study, we characterized *rap-1a* genes in the *B. divergens*-like phylogenetic group. The *rap-1a* genes belong to a multigene family, and multiple copies have already been demonstrated in a few *Babesia* species: two copies in *B. bovis* and *B. canis*, four to five in *B. ovis*, at least seven copies in *Babesia* sp. Xinjiang, eleven in *B. bigemina* and twelve in the *B. motasi*-like group members [52,56,57,58,59,60,61]. The multiple copies of *rap-1a* are usually different, allowing their differentiation, except in the case of *B. divergens* where the presence of two identical copies was highlighted when its genome was sequenced [52]. It is highly probable that two identical and therefore undistinguishable copies of *rap-1a* exist in all the *B. divergens* isolates characterized. We cannot exclude the presence of two identical copies also for members of the *Babesia* sp. MO1-like clade. In all *B. capreoli* isolates analyzed, two different but closely related *rap-1a* copies, named *rap-1a1* and *rap-1a2* (sequence identities of about 96%) were identified. Each copy is equally different from either *B. divergens*, *Babesia* sp. FR1 or *Babesia* sp. AR1 *rap-1a* genes, and the two copies place as sister groups to each other, indicating a gene duplication that occurred after *B. capreoli* speciation. While genetic divergence occurred between the two *rap-1a* copies of *B. capreoli*, it was not the case between *B. divergens rap-1a* copies. *B. divergens rap-1a* gene has been previously characterized and its genetic variability among cattle and human isolates was found to be limited [51,62,63]. A greater sequence diversity among *B. capreoli* isolates compared to *B. divergens* was also highlighted in the case of the merozoite surface antigen *Bc37/41* compared to *Bd37*, and a greater selection pressure was hypothesized [64]. This could also explain the sequence divergence between the two copies of *rap-1a* in *B. capreoli* and not between the two *B. divergens* copies of *rap-1*. But a more recent event of the *rap-1a* gene duplication in *B. divergens* could also explain the difference in sequence divergence between the two copies. Whether the last common ancestor of members of the *B. divergens*-like group possesses one or two copies of *rap-1a*, or when and how many times *rap-1a* gene duplication occurred in the speciation process is difficult to evaluate. Despite the presence of all motives that characterized RAP-1 family members, *rap-1b* sequence identity with the other two copies of *rap-1a* in the *B. divergens* genome is extremely low (45%) and was not amplified with the primers used.

In conclusion, we describe here a case of human babesiosis in Europe (France) due to a *Babesia* isolate more closely related to the American *Babesia* sp. MO1 and AR1 than to *B. divergens*. Using two discriminant molecular markers, our study confirms the existence of three phylogenetic clades within the *B. divergens*-like group that would deserve the rank of species as their phylogenetic classification corresponds with their natural hosts; *B. divergens* natural host is cattle, *B. capreoli* infects mainly roe deer, and *Babesia* sp. MO1-like parasites probably infect Laporidae. 

In Europe, diagnosis of human babesiosis is complicated due to its infrequency which often leads to a delayed detection and treatment. This delayed treatment promotes the development of a fulminant manifestation of this parasitic disease, in particular for asplenic or immunocompromised patients. Thus, the infection results often in the death of the patient, which could probably have been avoided by a more precocious diagnosis and treatment [23,26]. In Europe, the serological and molecular tools developed to diagnose *B. divergens* infections should principally be adequate to detect *Babesia* sp. FR1 infections. However, it needs to be taken into account that atypical immunofluorescence patterns (dots and weak fluorescence of the parasite surface) may lead to a negative conclusion when carrying out an immunofluorescence test. Prophylactic treatments are advised, such as wearing long clothes and performing skin examination for tick detection after exposure to high-risk environments.

## 4. Materials and Methods

### 4.1. Babesia Isolates and DNA Origins 

A preliminary identification of *Babesia* sp. FR1 responsible for the mild form of babesiosis was performed on blood smears stained with May-Grünwald Giemsa. Further diagnosis was carried out by serology (IFAT with *B. divergens* antigen) [31], as well as 18S rRNA gene amplification [33] and sequencing from blood DNA extracted using the Nucleospin Blood kit according to the manufacturer’s instructions (Macherey-Nagel, Düren, Germany).

*B. capreoli* isolates were collected and characterized in previous studies performed at our lab [33,47]. We included in our analysis four in vitro cultivated isolates from roe deer blood samples or spleen, from three different regions of France (Supplementary Table S1). *B. divergens* isolates were also cultivated in vitro from acute piroplasmosis cases in cows (11 isolates) or in humans (one isolate) [55]. 

DNA from cultured *B. capreoli* clonal lines (2704C, 2770 F6, 2801 F10 and CVD08 005) was extracted as previously mentioned. 

DNA from one American case of human babesiosis was kindly provided by Mayo Medical Laboratory, Rochester, USA. This fatal case occurred in an asplenic patient, in Arkansas in 2015, with a possible acquisition through transfusion [35]. Due to its geographical origin, we named this isolate *Babesia* sp. AR1. It was characterized as a *Babesia* sp. MO1-like babesiosis etiological agent.

As all these isolates are very closely related to *B. divergens*, we have used throughout the manuscript the terminology *B. divergens*-like phylogenetic group to qualify the following isolates, species or clades: *B. divergens*, *B. capreoli*, *Babesia* sp. MO1, *Babesia* sp. AR1, and the new *Babesia* isolate, named *Babesia* sp. FR1.

### 4.2. Comparison of 18S rDNA Sequences within the B. divergens Taxonomic Group

*Babesia* sp. AR1 18S rRNA sequences (560 bp) from the previously mentioned isolates were kindly provided by Mayo Medical Laboratory, Rochester, USA. Published *B. capreoli* as well as *B. divergens* 18S rRNA sequences were used as a comparison [33]. Their origin and accession numbers are described in Supplementary Table S1. The partial 18S rRNA sequence of *Babesia* sp. FR1 was obtained using the same primers [33] (Table 4). With the aim of obtaining sequences of comparable sizes, the 18S rRNA partial sequence of *Babesia* sp. AR1 was also amplified with these primers and sequenced. The alignment was done using the ClustalW program as implemented in the Geneious R6 software (https://www.geneious.com accessed on 1 October 2021).

### 4.3. Amplification of Ama-1 (Apical Membrane Antigen-1) and Rap-1a (Rhoptry Associated protein-1) Genes for B. capreoli, Babesia *sp*. AR1, and Babesia *sp*. FR1

PCR was performed to amplify the *ama-1* and *rap-1a* genes of *B. capreoli*, *Babesia sp.* AR1, and *Babesia* sp. FR1 isolates using ama1-S1/ama1-R3 and rap1-fw/rap1-rev primers respectively (Table 4). Reactions were carried out in 30 µL reaction mixtures containing 1 X GoTaq buffer, 4 mM MgCl_2_, 0.2 mM of each dNTP (Eurobio Scientific, Les Ulis, France), 1 unit GoTaq G2 Flexi DNA Polymerase (Promega, Madison, WI, USA), 0.5 µM of each primer and 1 µL of DNA template. The amplification conditions comprised 5 min at 95 °C followed by 40 cycles of 30 s at 95 °C, 30 s at the temperatures indicated in Table 4, 1 min 30 s at 72 °C, and a final extension at 72 °C for 5 min. The amplified fragments were purified with the ExoSAP-IT reagent according to the manufacturer’s instructions (Affymetrix, Santa Clara, CA, USA) and sequencing was performed on both strands (Eurofins Genomics, Ebersberg, Germany) using the same primers for *rap-1a* gene, or using primers distributed along the sequence for *ama-1* gene (Table 4). Sequences were then assembled using the Geneious R6 software.

For the four isolates of *B. capreoli* (2704C, 2770 F6, 2801 F10 and CVD08 005), preliminary sequencing results of *rap-1a* gene highlighted superposed chromatograms at the gene 3′ end, suggesting the presence of multiple copies of this gene, a frequent feature for this gene. The PCR products were therefore cloned in the pGEM-T easy vector according to the manufacturer’s instructions (Promega, Madison, WI, USA), to determine the number and sequences of the putative different copies of the *rap-1a* gene. *Escherichia coli* strain BL21 was transformed with the plasmid constructions and colonies with the expected inserts were selected by direct colony PCR using vector primers: T7 and SP6. Recombinant plasmids were then isolated using the Nucleospin Plasmid kit (Macherey-Nagel, Düren, Germany) and both strands of the inserts were sequenced using vector primers (Eurofins, Genomics, Ebersberg, Germany). Then primers were designed to selectively amplify the different *rap-1a* gene copies of *B. capreoli* isolates (Table 4). Primers rap1-a1-fw or rap1-a2-fw were associated with primer rap1-rev to amplify the 3′ part of respective *rap-1a* copies. Primers rap1-a1-rev or rap1-a2-rev were associated with primer rap1-fw to amplify the 5′ part of *rap-1a* copies (same reaction and cycling conditions as above). PCR products were purified and sequenced as already mentioned. 

### 4.4. Comparison of Ama-1 and Rap-1a DNA Sequences for B. divergens-Like Phylogenetic Group Members

The resulting *ama-1* and *rap-1a* DNA sequences of *B. capreoli*, *Babesia* sp. AR1 and *Babesia* sp. FR1 were aligned along with the published *ama-1* and *rap-1a* DNA sequences of *B. divergens* isolates using the ClustalW program as implemented in the Geneious R6 software.

### 4.5. Phylogenetic Analysis 

Phylogenetic relationships within the *Babesia divergens*-like group were inferred using published sequences available in GenBank (Supplementary Table S1) and sequence data produced in the present study (18S rRNA, *ama-1* and *rap-1a* sequences). Sequences were aligned using Muscle as implemented in MEGA version X [65]. Phylogenetic analyses used a trimmed alignment of 1189 bp with complete deletion option for the 18S rRNA gene, 1728 bp and 1138 bp with complete deletion option for the *ama-1* and *rap-1a* coding sequences respectively. Maximum likelihood phylogenetic trees were produced using MEGA-X, with 1000 bootstrap replications based on the Tamura 3-parameter model [66] for the 18S rRNA gene and the *ama-1* gene, and based on the Kimura 2-parameter model [67] model for *rap-1a* gene. For the *ama-1* and *rap-1a* sequences, the three codon positions were included. The appropriate model of nucleotide substitution for ML analysis was selected based on the Bayesian Information Criterion (BIC) computed by MEGA-X.

### 4.6. Genbank Deposition 

Nucleotidic sequences obtained in this study were submitted to GenBank with accession numbers MZ825347 and OK086051 for the 18S rRNA sequence of *Babesia* sp. FR1 and *Babesia* sp. AR1 respectively, MZ836259 and MZ836261 for the *ama-1* sequences of *Babesia* sp. AR1 and *Babesia* sp. FR1 respectively, MZ836258 and MZ836260 for the *rap-1a* sequences of *Babesia* sp. AR1 and *Babesia* sp. FR1 respectively. Accession numbers for *rap-1a* and *ama-1* sequences of *B. capreoli* are presented in Supplementary Table S1.

## Figures and Tables

**Table 1:** Biological and molecular features of the *Babesia* belonging to the *B. divergens*-like phylogenetic group.

Organism	Natural Host	Human Infection	Geographical Occurence	Vector	18S rRNA Sequence Differences at Nucleotide Position ^b^			
					631	663	819	1637
*B. divergens*	Cattle	+	Europe	*I. ricinus*	A	A	T	C
*B. capreoli*	Roe deer	−	Europe	*I. ricinus*	G	T	T	T
*Babesia* sp. MO1/AR1	Cottontail rabbit	+	USA	*I. dentatus* ^a^	G	A	A	T
*Babesia* sp. FR1	nd ^c^	+	France	nd ^c^	G	A	T	T

^a^ likely vector according to [36]. ^b^ corresponding to position described in [33]. ^c^ not determined.

**Table 2:** Genetic variability of 18S rRNA, *ama-1* and *rap-1a* genes within *B. divergens* and *B. capreoli*.

*Babesia* Species	Gene	Number of Isolates	Nucleotide Differences	Identities
*B. divergens*	18S rRNA	12	None	100% [33]
	*rap-1a*	12	0–6 nt/1242 bp	99.6–100% [46]
	*ama-1*	9	0–2 nt/1821 bp	99.9–100% [45]
*B. capreoli*	18S rRNA	9	None	100% [33]
	*rap-1a1*	4	0–7 nt/1236 bp	99.5–100%
	*rap-1a2*	4	0–5 nt/1203 bp	99.6–100%
	*ama-1*	4	0–6 nt/1821 bp	99.7–100%

**Table 3:** Contingency table for 18S rRNA, *rap-1a* (partial cds) and *ama-1* genes. For the 18S rRNA sequences, the number of nucleotide differences between 18S rRNA gene sequences are indicated in red, instead of the identity percentages. For *ama-1* and *rap-1a* genes, percentage of identities are indicated in green and blue, respectively. Sequence identities with each *rap-1a* copy (*rap-1a1* and *rap-1a2*) are indicated. The identities are calculated excluding the deletions and duplication indicated in Figure 2. Accession numbers of sequences used to perform the analysis are indicated in supplementary Table S1.

Organism	*B. divergens*	*B. capreoli*	*Babesia* sp. AR1	*Babesia* sp. FR1
*B. divergens*	**0**			
	**99.9–100%**			
	**99.6–100%**			
*B. capreoli*	**3**	**0**		
	**95–95.3%**	**99.7–100%**		
	**(rap-1a1) 86.6–89.1%**	**(rap-1a1) 99.5–100%**		
	**(rap1-a2) 88.6–89.1%**	**(rap1-a2) 99.6–100%**		
*Babesia* sp. AR1	**3**	**2**	**0**	
	**94.3%**	**97.2–97.3%**	**100%**	
	**89.5–89.8%**	**(rap-1a1) 95.1–95.4%**	**100%**	
		**(rap1-a2) 95.3–95.7%**		
*Babesia* sp. FR1	**2**	**1**	**1**	**0**
	**94.5%**	**97.3–97.4%**	**98.7%**	**100%**
	**89.8–90%**	**(rap-1a1) 95.2–95.5%**	**98.7%**	**100%**
		**(rap1-a2) 95.7–96.1%**		

**Table 4:** Description of the primers used in this study for gene amplification as well as sequencing.

Target Gene	Primer Name	Sequence (5′–3′)	Tm (°C)	PCR	Amplicon Length (bp)	Sequencing	References
18S rRNA	CRYPTOF	AACCTGGTTGATCCTGCCAGTAGTCAT	63	×	1728	×	[33]
	CRYPTOR	TGATCCTTCTGCAGGTTCACCTA		×		×	
	BAB-GF2	GTCTTGTAATTGGAATGATGG	61	×	560	×	[11]
	BAB-GR2	CCAAAGACTTTGATTTCTCT		×		×	
*ama-1*	ama1-S1	TGACTGCCATATCGACGAAG	61	×	≈2000	×	this study
	ama1-R3	CTCTAGTGAATTACGATAGC		×		×	[50]
	ama1-As1	GGCGGATATTCGGTTGAGG				×	this study
	ama1-S2	CATGGCCAAGTTTGACCTTG				×	this study
	ama1-As2	CTGCGTCACGCGTGAATTC				×	this study
	ama1-S3	CTCCTGTGTATGGAGCCGA				×	this study
	ama1-As3	GTGAAAGCGCGGTTGTGAC				×	this study
	ama1-S4	AGCAGTTGGATCGCCTCTC				×	this study
*rap-1a*	rap1-fw	AATGTCCTACTGGGAAACGC	58	×	≈1300	×	this study
	rap1-rev	GCGGAGTCCATGCCTGTACC		×		×	this study
*rap-1a1* (5′)	rap1-fw	see above	58	×	1146	×	
	rap1-a1-rev	GCTTAGTAGCATGCATCTTC		×		×	this study
*rap-1a1* (3′)	rap1-a1-fw	GGACTCCGAGAAAAAGGATG	58	×	261	×	this study
	rap1-rev	see above		×		×	
*rap-1a2* (5′)	rap1-fw	see above	58	×	1123	×	
	rap1-a2-rev	TGGAACAACTTCTTCATAGG		×		×	this study
*rap-1a2* (3′)	rap1-a2-fw	GGGCTTCTGGAAAAAGAAGG	58	×	228	×	this study
	rap1-rev	see above		×		×	

## Supplementary Materials

The following are available online at https://www.mdpi.com/article/10.3390/pathogens10111433/s1, Table S1: Description of *B. divergens* (A) and *B. capreoli* (B) clonal lines used in the study and Genbank accession numbers. Figure S1: (A) Major differences in *ama-1* gene and AMA-1 protein sequences. (B) Major differences in *rap-1a* gene and RAP-1A protein sequences.

## References

[1] Schnittger L., Rodriguez A.E., Florin-Christensen M., Morrison D.A. (2012). *Babesia*: A world emerging. Infect. Genet. Evol..

[2] Herwaldt B.L., de Bruyn G., Pieniazek N.J., Homer M., Lofy K.H., Slemenda S.B., Fritsche T.R., Persing D.H., Limaye A.P. (2004). *Babesia divergens*-like infection, Washington State. Emerg. Infect. Dis..

[3] Herwaldt B., Persing D.H., Précigout E.A., Goff W.L., Mathiesen D.A., Taylor P.W., Eberhard M.L., Gorenflot A.F. (1996). A fatal case of babesiosis in Missouri: Identification of another piroplasm that infects humans. Ann. Intern. Med..

[4] Vannier E.G., Diuk-Wasser M.A., Ben Mamoun C., Krause P.J. (2015). Babesiosis. Infect. Dis. Clin. N. Am..

[5] Jiang J.F., Zheng Y.C., Jiang R.R., Li H., Huo Q.B., Jiang B.G., Sun Y., Jia N., Wang Y.W., Ma L. (2015). Epidemiological, clinical, and laboratory characteristics of 48 cases of “*Babesia venatorum*” infection in China: A descriptive study. Lancet Infect. Dis..

[6] Wang J., Zhang S., Yang J., Liu J., Zhang D., Li Y., Luo J., Guan G., Yin H. (2019). *Babesia divergens* in human in Gansu province, China. Emerg. Microbes Infect..

[7] Jia N., Zheng Y.C., Jiang J.F., Jiang R.R., Jiang B.G., Wei R., Liu H.B., Huo Q.B., Sun Y., Chu Y.L. (2018). Human babesiosis caused by a *Babesia crassa*-like pathogen: A case series. Clin. Infect. Dis..

[8] Skrabalo Z., Deanovic Z. (1957). Piroplasmosis in man; report of a case. Doc. Med. Geogr. Trop..

[9] Gray J.S. (2006). Identity of the causal agents of human babesiosis in Europe. Int. J. Med. Microbiol..

[10] Uguen C., Girard L., Brasseur P., Leblay R. (1997). Human babesiosis in 1997. Rev. Med. Interne.

[11] Herwaldt B.L., Cacciò S., Gherlinzoni F., Aspöck H., Slemenda S.B., Piccaluga P., Martinelli G., Edelhofer R., Hollenstein U., Poletti G. (2003). Molecular characterization of a non-*Babesia divergens* organism causing zoonotic babesiosis in Europe. Emerg. Infect. Dis..

[12] Häselbarth K., Tenter A.M., Brade V., Krieger G., Hunfeld K.P. (2007). First case of human babesiosis in Germany—Clinical presentation and molecular characterisation of the pathogen. Int. J. Med. Microbiol..

[13] Bläckberg J., Lazarevic V.L., Hunfeld K.P., Persson K.E.M. (2018). Low-virulent *Babesia venatorum* infection masquerading as hemophagocytic syndrome. Ann. Hematol..

[14] Stahl P., Poinsignon Y., Pouedras P., Ciubotaru V., Berry L., Emu B., Krause P.J., Ben Mamoun C., Cornillot E. (2018). Case report of the patient source of the *Babesia microti* R1 reference strain and implications for travelers. J. Travel. Med..

[15] Hildebrandt A., Hunfeld K.P., Baier M., Krumbholz A., Sachse S., Lorenzen T., Kiehntopf M., Fricke H.J., Straube E. (2007). First confirmed autochthonous case of human *Babesia microti* infection in Europe. Eur. J. Clin. Microbiol. Infect. Dis..

[16] Centeno-Lima S., do Rosário V., Parreira R., Maia A.J., Freudenthal A.M., Nijhof A.M., Jongejan F. (2003). A fatal case of human babesiosis in Portugal: Molecular and phylogenetic analysis. Trop. Med. Int. Health..

[17] Corpelet C., Vacher P., Coudore F., Laurichesse H., Conort N., Souweine B. (2005). Role of quinine in life-threatening *Babesia divergens* infection successfully treated with clindamycin. Eur. J. Clin. Microbiol. Infect. Dis..

[18] Haapasalo K., Suomalainen P., Sukura A., Siikamaki H., Jokiranta T.S. (2010). Fatal babesiosis in man, Finland, 2004. Emerg. Infect. Dis..

[19] Gonzalez L.M., Rojo S., Gonzalez-Camacho F., Luque D., Lobo C.A., Montero E. (2014). Severe babesiosis in immunocompetent man, Spain, 2011. Emerg. Infect. Dis..

[20] González L.M., Castro E., Lobo C.A., Richart A., Ramiro R., González-Camacho F., Luque D., Velasco A.C., Montero E. (2015). First report of *Babesia divergens* infection in an HIV patient. Int. J. Infect. Dis..

[21] Tanyel E., Guler N., Hokelek M., Ulger F., Sunbul M. (2015). A case of severe babesiosis treated successfully with exchange transfusion. Int. J. Infect. Dis..

[22] O’Connell S., Lyons C., Abdou M., Patowary R., Aslam S., Kinsella N., Zintl A., Hunfeld K.P., Wormser G.P., Gray J. (2017). Splenic dysfunction from celiac disease resulting in severe babesiosis. Ticks Tick Borne Dis..

[23] Asensi V., González L.M., Fernández-Suárez J., Sevilla E., Navascués R.Á., Suárez M.L., Lauret M.E., Bernardo A., Carton J.A., Montero E. (2018). A fatal case of *Babesia divergens* infection in Northwestern Spain. Ticks Tick Borne Dis..

[24] Kukina I.V., Zelya O.P., Guzeeva T.M., Karan L.S., Perkovskaya I.A., Tymoshenko N.I., Guzeeva M.V. (2019). Severe babesiosis caused by *Babesia divergens* in a host with intact spleen, Russia, 2018. Ticks Tick Borne Dis..

[25] Mørch K., Holmaas G., Frolander P.S., Kristoffersen E.K. (2015). Severe human *Babesia divergens* infection in Norway. Int. J. Infect. Dis..

[26] Kukina I.V., Guzeeva T.M., Zelya O.P., Ganushkina L.A. (2018). Fatal human babesiosis caused by *Babesia divergens* in an asplenic host. IDCases.

[27] Strizova Z., Havlova K., Patek O., Smrz D., Bartunkova J. (2020). The first human case of babesiosis mimicking Reiter’s syndrome. Folia Parasitol..

[28] Loutan L., Rossier J., Zufferey G., Cuénod D., Hatz C., Marti H.P., Gern L. (1994). Human babesiosis: First case report in Switzerland. Rev. Med. Suisse Romande.

[29] Martinot M., Zadeh M.M., Hansmann Y., Grawey I., Christmann D., Aguillon S., Jouglin M., Chauvin A., De Briel D. (2011). Babesiosis in immunocompetent patients, Europe. Emerg. Infect. Dis..

[30] Paleau A., Candolfi E., Souply L., De Briel D., Delarbre J.M., Lipsker D., Jouglin M., Malandrin L., Hansmann Y., Martinot M. (2020). Human babesiosis in Alsace. Med. Mal. Infect..

[31] Lempereur L., Shiels B., Heyman P., Moreau E., Saegerman C., Losson B., Malandrin L. (2015). A retrospective serological survey on human babesiosis in Belgium. Clin. Microbiol. Infect..

[32] Duh D., Petrovec M., Bidovec A., Avsic-Zupanc T. (2005). Cervids as Babesiae hosts, Slovenia. Emerg. Infect. Dis..

[33] Malandrin L., Jouglin M., Sun Y., Brisseau N., Chauvin A. (2010). Redescription of *Babesia capreoli* (Enigk and Friedhoff, 1962) from roe deer (*Capreolus capreolus*): Isolation, cultivation, host specificity, molecular characterisation and differentiation from *Babesia divergens*. Int. J. Parasitol..

[34] Beattie J.F., Michelson M.L., Holman P.J. (2002). Acute babesiosis caused by *Babesia divergens* in a resident of Kentucky. N. Engl. J. Med..

[35] Burgess M.J., Rosenbaum E.R., Pritt B.S., Haselow D.T., Ferren K.M., Alzghoul B.N., Rico J.C., Sloan L.M., Ramanan P., Purushothaman R. (2017). Possible transfusion-transmitted *Babesia divergens*-like/MO1 infection in an Arkansas patient. Clin. Infect. Dis..

[36] Herc E., Pritt B., Huizenga T., Douce R., Hysell M., Newton D., Sidge J., Losman E., Sherbeck J., Kaul D.R. (2018). Probable locally acquired *Babesia divergens*-like infection in woman, Michigan, USA. Emerg. Infect. Dis..

[37] Goethert H.K., Telford S.R. (2003). Enzootic transmission of *Babesia divergens* among cottontail rabbits on Nantucket Island, Massachusetts. Am. J. Trop. Med. Hyg..

[38] Holman P.J., Spencer A.M., Droleskey R.E., Goethert H.K., Telford S.R. (2005). In vitro cultivation of a zoonotic *Babesia* sp. isolated from eastern cottontail rabbits (*Sylvilagus floridanus*) on Nantucket Island, Massachusetts. J. Clin. Microbiol..

[39] Holman P.J., Spencer A.M., Telford S.R., Goethert H.K., Allen A.J., Knowles D.P., Goff W.L. (2005). Comparative infectivity of *Babesia divergens* and a zoonotic *Babesia divergens*-like parasite in cattle. Am. J. Trop. Med. Hyg..

[40] Spencer A.M., Goethert H.K., Telford S.R., Holman P.J. (2006). In vitro host erythrocyte specificity and differential morphology of *Babesia divergens* and a zoonotic *Babesia* sp. from eastern cottontail rabbits (*Sylvilagus floridanus*). J. Parasitol..

[41] Holman P.J. (2006). Phylogenetic and biologic evidence that *Babesia divergens* is not endemic in the United States. Ann. N. Y. Acad. Sci..

[42] Hildebrandt A., Gray J.S., Hunfeld K.P. (2013). Human babesiosis in Europe: What clinicians need to know. Infection.

[43] Hildebrandt A., Zintl A., Montero E., Hunfeld K.P., Gray J. (2021). Human Babesiosis in Europe. Pathogens.

[44] Jahfari S., Hofhuis A., Fonville M., van der Giessen J., van Pelt W., Sprong H. (2016). Molecular Detection of tick-borne pathogens in humans with tick bites and erythema migrans, in the Netherlands. PLoS Negl. Trop. Dis..

[45] Moreau E., Bonsergent C., Al Dybiat I., Gonzalez L.M., Lobo C.A., Montero E., Malandrin L. (2015). *Babesia divergens* apical membrane antigen-1 (BdAMA-1): A poorly polymorphic protein that induces a weak and late immune response. Exp. Parasitol..

[46] Sun Y. (2010). Caractérisation moléculaire, localisation cellulaire et conservation des protéines impliquées dans le processus d’invasion des érythrocytes par *Babesia divergens*. Ph.D. Thesis.

[47] Bastian S., Jouglin M., Brisseau N., Malandrin L., Klegou G., L’Hostis M., Chauvin A. (2012). Antibody prevalence and molecular identification of *Babesia* spp. in roe deer in France. J. Wildl. Dis..

[48] Krause P.J. (2019). Human babesiosis. Int. J. Parasitol..

[49] Gorenflot A., Moubri K., Precigout E., Carcy B., Schetters T.P. (1998). Human babesiosis. Ann. Trop. Med. Parasitol..

[50] L’Hostis M., Chauvin A., Valentin A., Marchand A., Gorenflot A. (1995). Large scale survey of bovine babesiosis due to *Babesia divergens* in France. Vet. Rec..

[51] Agoulon A., Malandrin L., Lepigeon F., Vénisse M., Bonnet S., Becker C.A., Hoch T., Bastian S., Plantard O., Beaudeau F. (2012). A Vegetation Index qualifying pasture edges is related to *Ixodes ricinus* density and to *Babesia divergens* seroprevalence in dairy cattle herds. Vet. Parasitol..

[52] González L.M., Estrada K., Grande R., Jiménez-Jacinto V., Vega-Alvarado L., Sevilla E., Barrera J., Cuesta I., Zaballos Á., Bautista J.M. (2019). Comparative and functional genomics of the protozoan parasite *Babesia divergens* highlighting the invasion and egress processes. PLoS Negl. Trop. Dis..

[53] Zamoto-Niikura A., Tsuji M., Imaoka K., Kimura M., Morikawa S., Holman P.J., Hirata H., Ishihara C. (2014). Sika deer carrying *Babesia* parasites closely related to *B. divergens*, Japan. Emerg. Infect. Dis..

[54] Zamoto-Niikura A., Tsuji M., Qiang W., Morikawa S., Hanaki K.I., Holman P.J., Ishihara C. (2018). The *Babesia divergens* Asia lineage is maintained through enzootic cycles between *Ixodes persulcatus* and sika deer in Hokkaido, Japan. Appl. Environ. Microbiol..

[55] Malandrin L., L’Hostis M., Chauvin A. (2004). Isolation of *Babesia divergens* from carrier cattle blood using in vitro culture. Vet. Res..

[56] Dalrymple B.P., Casu R.E., Peters J.M., Dimmock C.M., Gale K.R., Böse R., Wright I.G. (1993). Characterisation of a family of multi-copy genes encoding rhoptry protein homologues in *Babesia bovis*, *Babesia ovis* and *Babesia canis*. Mol. Biochem. Parasitol..

[57] Suarez C.E., Palmer G.H., Hötzel I., McElwain T.F. (1998). Structure, sequence, and transcriptional analysis of the *Babesia bovis rap-1* multigene locus. Mol. Biochem. Parasitol..

[58] Suarez C.E., Palmer G.H., Florin-Christensen M., Hines S.A., Hötzel I., McElwain T.F. (2003). Organization transcription, and expression of rhoptry associated protein genes in the *Babesia bigemina rap-1* locus. Mol. Biochem. Parasitol..

[59] Niu Q., Bonsergent C., Guan G., Yin H., Malandrin L. (2013). Sequence and organization of the rhoptry-associated-protein-1 (*rap-1*) locus for the sheep hemoprotozoan *Babesia* sp. BQ1 Lintan (*B. motasi* phylogenetic group). Vet. Parasitol..

[60] Niu Q., Valentin C., Bonsergent C., Malandrin L. (2014). Strong conservation of rhoptry-associated-protein-1 (RAP-1) locus organization and sequence among *Babesia* isolates infecting sheep from China (*Babesia motasi*-like phylogenetic group). Infect. Genet. Evol..

[61] Niu Q., Marchand J., Yang C., Bonsergent C., Guan G., Yin H., Malandrin L. (2015). Rhoptry-associated protein (*rap-1*) genes in the sheep pathogen *Babesia* sp. Xinjiang: Multiple transcribed copies differing by 3′ end repeated sequences. Vet. Parasitol..

[62] Skuce P.J., Mallon T.R., Taylor S.M. (1996). Molecular cloning of a putative rhoptry associated protein homologue from *Babesia divergens*. Mol. Biochem. Parasitol..

[63] Rodriguez M., Alhassan A., Ord R.L., Cursino-Santos J.R., Singh M., Gray J., Lobo C.A. (2014). Identification and characterization of the RouenBd1987 *Babesia divergens* Rhopty-Associated Protein 1. PLoS ONE.

[64] Sun Y., Jouglin M., Bastian S., Chauvin A., Malandrin L. (2011). Molecular cloning and genetic polymorphism of *Babesia capreoli* gene Bcp37/41, an ortholog of *Babesia divergens* merozoite surface antigen Bd37. Vet. Parasitol..

[65] Kumar S., Stecher G., Li M., Knyaz C., Tamura K. (2018). MEGA X: Molecular evolutionary genetics analysis across computing platforms. Mol. Biol. Evol..

[66] Tamura K. (1992). Estimation of the number of nucleotide substitutions when there are strong transition-transversion and G + C-content biases. Mol. Biol. Evol..

[67] Kimura M. (1980). A simple method for estimating evolutionary rate of base substitutions through comparative studies of nucleotide sequences. J. Mol. Evol..

