# Supplementary material for: The New Human Babesia sp. FR1 Is a European Member of the Babesia sp. MO1 Clade
Source: Pathogens. 2021 Nov 4;10(11):1433. doi: 10.3390/pathogens10111433 (PMC8618789; doi:10.3390/pathogens10111433)
Supplement: Supplementary file 1 [file pathogens-10-01433-s001.zip › Figure S1.pdf]

Figure S1. (A) Major differences in *ama-1* gene and AMA-1 protein sequences. (B) Major differences in *rap-1* gene and RAP-1 protein sequences.

(A)

18 bp acid nucleic deletion

```
                                   553               570
                                    |                 |
B. divergens ama-1      AACGCAAGGAATCGTGCGGCGGCCGAGAAAACTGAGATTATTCTATCGCCTGTGTCCGCA 600
B. capreoli ama-1       ACCGCAAGGAATCCTGCGGCGGCCGAGAAAACTGCGATTACTCTATCGCCTGTGTCCGCA 600
Babesia sp.FR1 ama-1    TACGCAAGGAATCCTGCGGGGGCCATAAAAACTGCGATTACTCTATCGCCTGTGTCCGCA 600
Babesia sp.AR1 ama-1    AACGCAAGGAAT------------------ACTGCGATTACTCTATCGCCTGTGTCCGCA 582
                          **********                  **** ***** *******************
```

corresponding amino-acid sequence

```
B. divergens AMA-1       NARNRAAAEKTEIILSPVSA
B. capreoli AMA-1        TARNPAAAEKTAITLSPVSA
Babesia sp. FR1 AMA-1    YARNPAGAIKTAITLSPVSA
Babesia sp. AR1 AMA-1    NARN------TAITLSPVSA
                          ***      * * ******
```

36 bp acid nucleic duplication

```
                                                             1496                                1531
                                                               |                                   |
B. divergens ama-1    TACTACTATTGCTCAGAAGAGAAGCCTTCATCCTGG------------------------------------GC
B. capreoli ama-1     TACTACTATTGCTCGCAAGAGAAACCTACATCATGG------------------------------------GC
Babesia sp.FR1 ama-1  TACTACTATTGCTCGCAAGAGAAGCCTCCATCCTGGGACTACTATCGCTCGCAAGAGAAGCCTCCATCCTGGGC
Babesia sp. AR1 ama-1 TACTACTATTGCTCGCAAGAGAAGCCTCCATCCTGG------------------------------------GC
                      **************  ******* *** **** ***                                    **
```

corresponding amino-acid sequence

```
B. divergens AMA-1       YYYCSEEKPSSW------------A
B. capreoli AMA-1        YYYCSQEKPTSW------------A
Babesia sp. FR1 AMA-1    YYYCSQEKPPSWDYYRSQEKPPSWA
Babesia sp. AR1 AMA-1    YYYCSQEKPPSW------------A
                         *****:*** **            *
```

(B)

3 bp and 33 bp acid nucleic deletions

```
                                                                                1012 1014
                                                                                  |  |
B. divergens rap-1      TTGTTCAGCAAGCGTGCTCCATCYTCTTCTACTGAGGGGCCATGGCACAAGAAGGTTACT
Babesia sp. FR1 rap-1   TTGTTCAGCAAGCGCCCTTCATCTTCTTCTACTGAGGGGTTCTGGAAAAAGAAGGTTACT
Babesia sp. AR1 rap-1   TTGTTCAGCAAGCGCCCTTCATCTTCTTCTACTGAGGCATCCTGGAAAAAG---GTTACT
B. capreoli rap-1a1     TTGTTCAGCAAGCGCCCTTCATCTACTTCTACTGAGGACTCCGAGAAAAAGRATGTTACT
B. capreoli rap-1a2     TTGTTCARCAAGCGCCCTTCATCTACTTCTACTGAGGRSTTCTGGAAAAAGAAGGTTACT
                        ******* ******  ** ****  ************       * * ***   ******

                                1030                            1062
                                  |                               |
B. divergens rap-1      CACTCTGTG---------------------------------AAGAAGATGTTAAACGAG
Babesia sp. FR1 rap-1   CAACCTGTGAAGGATTATTTAAACAAGAAGATGCATGCTACTAAGCAGTTGTTTCACGAG
Babesia sp. AR1 rap-1   CAACCTGTGAAGGATTATTTAAACAAGAAGATGCATGCTACTAAGCAGTTGTTTCACGAG
B. capreoli rap-1a1     CAACCTATGAAGGATTATTTAAACAAGAAGATGCATGCTACTAAGCAGTTGTTCCATGAG
B. capreoli rap-1a2     CAACCTATG---------------------------------AAGAAGTTGTTCCACGAG
                        **  ** **                                 *** ** ****  * ***
```

corresponding amino-acid sequence

```
B. divergens RAP-1       APSSSTEGPWHKKVTHSV-----------KKMLNE
Babesia sp. FR1 RAP-1    PSSSSTEGFWKKKVTQPVKDYLNKKMHATKQLFHE
Babesia sp. AR1 RAP-1    PSSSSTEASW-KKVTQPVKDYLNKKMHATKQLFHE
B. capreoli RAP-1a1      PSSTSTEDSEKKNVTQPMKDYLNKKMHATKQLFHE
B. capreoli RAP-1a2      PSSTSTEXFWKKKVTQPM-----------KKLFHE
```
