# Supplementary material for: The New Human Babesia sp. FR1 Is a European Member of the Babesia sp. MO1 Clade
Source: Pathogens. 2021 Nov 4;10(11):1433. doi: 10.3390/pathogens10111433 (PMC8618789; doi:10.3390/pathogens10111433)
Supplement: Supplementary file 1 [file pathogens-10-01433-s001.zip › Table S1.pdf]

Table S1

Description of *B. divergens* (A) and *B. capreoli* (B) clonal lines used in the study and Genbank accession numbers (new accession numbers are indicated in red).

A

Clonal line	French county origin	Host and isolation year	Gene accession numbers		
			18S rRNA[(1)]	*rap-1*	*ama-1*[(2)]
Rouen 87 F5	Seine-Maritime	Human - 1987	FJ944822	HQ538430	MZ836245
1406B F10	Calvados	Bovine		HQ538419	
1505B F14	Cantal			HQ538420	
1705A G10	Charente-Maritime			HQ538421	MZ836244
1802A G8	Cher	1988	FJ944825	HQ538422	
2705A E11	Eure			HQ538423	
3601B E2	Indre			HQ538424	
4201B D4	Loire	(n=11)		HQ538425	
4903A D11	Maine et Loire			HQ538426	
5012A G3	Manche			HQ538427	
7904A G11	Deux-Sèvres			HQ538428	
8706A E8	Haute-Vienne			HQ538429	

B

Isolate or clones	French county origin	Isolation year	Gene accession numbers			
			18S rRNA[(3)]	*rap-1a1*	*rap-1a2*	*ama-1*
2704C	Deux-Sèvres	2008		MZ836246	MZ836250	MZ836254
2770 F6	Marne	2008	FJ944827	MZ836247	MZ836251	MZ836255
2801 F10	Deux-Sèvres	2008	FJ944828	MZ836248	MZ836252	MZ836256
CVD 08-05	Cher	2008		MZ836249	MZ836253	MZ836257

[(1)]18S rRNA sequences are all identical within *B. divergens* clones.

[(2)] the *ama-1*sequence of clones 1505B F14 and 3601B E2 is identical to MZ836245 and the *ama-1* sequence from the other clones is identical to MZ836244.

[(3)] 18S rRNA sequences are all identical within *B. capreoli* clones.
